# EHMTI-0123. New-onset headaches heralding demyelination

**DOI:** 10.1186/1129-2377-15-S1-D33

**Published:** 2014-09-18

**Authors:** M Vinjam, M Khalil, A Al-Din

**Affiliations:** 1 Neurology Dept., Pinderfield's General Hospital, Wakefield, UK

## Background

Headache is a rare presenting demyelinating feature in people who go on to develop multiple sclerosis (MS). It has been suggested that approximately 2% of MS patients present with headache. There has been a debate whether MS patient group are at higher risk of developing headaches. We are not aware of any case reports where an occipital headache heralds first or recurrent high cervical cord demyelinating lesion.

## Aim

To report headache characteristics in patients whose headache was the main presenting symptom of MS

## Method

We prospectively collected data from patients who were referred to the MS clinic with new onset headaches who were found to have first demyelinating episode or MS relapse over the last 12 months. Magnetic Resonance Imaging (MRI) scans were reviewed for relevant demyelinating lesions.

## Results

We identified four patients with evidence of demyelination whose main symptom was new onset-headache (three with stabbing occipital headache and one with thunderclap headache) and were found to have relevant abnormal physical examination.

Of these four patients, three had demylinating plaque at C1-C2 segments. In 2 out of 4 cases, headache represented the first demyelinating episode.

## Conclusion

Cervical spinal cord is a heavily myelinated structure, demyelinating plaques could induce nociceptive inflow by affecting the trigemino-cervical complex.

It is the traditional thinking that headaches are an unlikely presentation of a demyelinating episode, however detailed headache history coupled with appropriate examination and investigations could potentially reveal conditions which requires totally different therapeutic approach from the common primary headache disorders.

No conflict of interest.

